# Bitter melon derived extracellular vesicles enhance the therapeutic effects and reduce the drug resistance of 5-fluorouracil on oral squamous cell carcinoma

**DOI:** 10.1186/s12951-021-00995-1

**Published:** 2021-08-28

**Authors:** Meng Yang, Qingqiong Luo, Xu Chen, Fuxiang Chen

**Affiliations:** 1 Department of Clinical Immunology, Ninth People’s Hospital, Shanghai Jiao Tong University School of Medicine, Shanghai, 200011 People’s Republic of China; 2 Faculty of Medical Laboratory Science, School of Medicine, Shanghai Jiao Tong University, Shanghai, 200025 People’s Republic of China

**Keywords:** Bitter melon, Extracellular vesicles, Oral squamous cell carcinoma, NLRP3, 5-Fluorouracil

## Abstract

**Background:**

Plant-derived extracellular vesicles (PDEVs) have been exploited for cancer treatment with several benefits. Bitter melon is cultivated as a vegetable and folk medicine with anticancer and anti-inflammatory activities. 5-Fluorouracil (5-FU) is widely used for cancer treatment. However, 5-FU-mediated NOD-like receptor family pyrin domain containing 3 (NLRP3) inflammation activation induced the resistance of oral squamous cell carcinoma (OSCC) cells to 5-FU. In this study, we explored the potential of bitter melon-derived extracellular vesicles (BMEVs) for enhancing the therapeutic efficacy and reduce the resistance of OSCC to 5-FU.

**Results:**

Herein, we demonstrate that bitter melon derived extracellular vesicles (BMEVs), in addition to their antitumor activity against OSCC have intrinsic anti-inflammatory functions. BMEVs induced S phase cell cycle arrest and apoptosis. Apoptosis induction was dependent on reactive oxygen species (ROS) production and JUN protein upregulation, since pretreatment with *N*-acetyl cysteine or catechin hydrate could prevent apoptosis and JUN accumulation, respectively. Surprisingly, BMEVs significantly downregulated NLRP3 expression, although ROS plays a central role in NLRP3 activation. We further assessed the underlying molecular mechanism and proposed that the RNAs of BMEVs, at least in part, mediate anti-inflammatory bioactivity. In our previous studies, NLRP3 activation contributed to the resistance of OSCC cells to 5-FU. Our data clearly indicate that BMEVs could exert a remarkable synergistic therapeutic effect of 5-FU against OSCC both in vitro and in vivo. Most notably, NLRP3 downregulation reduced the resistance of OSCC to 5-FU.

**Conclusions:**

Together, our findings demonstrate a novel approach to enhance the therapeutic efficacy and reduce the drug resistance of cancer cells to chemotherapeutic agents, which provides proof-of-concept evidence for the future development of PDEVs-enhanced therapy.

**Graphic Abstract:**

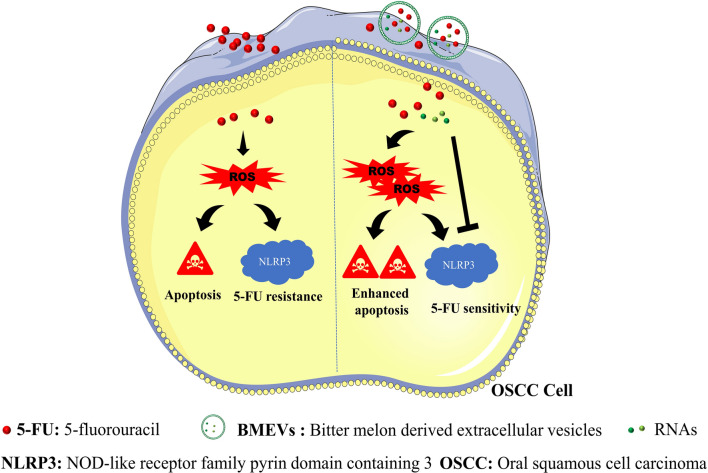

**Supplementary Information:**

The online version contains supplementary material available at 10.1186/s12951-021-00995-1.

## Background

Nanotechnology provides promising methods for drug delivery to overcome the limitations of conventional anticancer therapy [1]. Recently, edible plant-derived extracellular vesicles (PDEVs) have shown great potential as the drug delivery systems, because of their desirable morphology, environmental safety, and feasible large-scale preparation [2]. More importantly, PDEVs have intrinsic bioactivities. In our previous study, we showed that lemon derived EVs exerted tumor suppressor functions in gastric cancer [3]. Ginger- and mushroom-derived EVs downregulated the expression of NLRP3 (NLR family pyrin domain containing 3) [4, 5], indicating that PDEVs exhibit anti-inflammatory effects.

Oral squamous cell carcinoma (OSCC), with properties of rapid local invasion and a high recurrence rate, accounts for approximately 90% of malignant tumors in the oral cavity [6]. Despite advances in cancer treatment, the 5-year survival rate is only approximately 30% in OSCC with recurrence [7]. To a certain extent, the inherent or acquired resistance to chemotherapy agents of recurrent OSCC patients contributes to treatment failure [8]. 5-Fluorouracil (5-FU) is one of the commonly used chemotherapy drugs to treat OSCC [9]. However, its side effects and chemoresistance greatly limit its antitumor efficacy. Many studies have indicated that chemotherapy-induced inflammation may lead to drug resistance and metastasis [10]. Our previous studies showed that upregulation of the NLRP3 inflammasome induced by 5-FU contributed to drug resistance [11]. Thus, alternative strategies are urgently needed to enhance the therapeutic efficacy and reduce the resistance to chemotherapeutic agents.

Bitter melon has been cultivated as a vegetable, and has been used as a folk medicine with antitumor and anti-inflammatory effects [12]. The extract of bitter melon have exhibited antitumor effects on several kinds of cancers including OSCC [13, 14]. A previous study showed that freeze-dried bitter melon juice inhibited the expression of NLRP3 [15]. However, the bioactivities of bitter melon derived extracellular vesicles (BMEVs) have not yet been studied. As BMEVs are isolated from bitter melon juice, they may also have anti tumor and anti-inflammatory bioactivities.

Herein, we isolated BMEVs from bitter melon juice and assessed the synergistic therapeutic efficacy of BMEVs and 5-FU on OSCC. Our data demonstrated that BMEVs could suppress OSCC proliferation and induce apoptosis, which was mediated by the generation of reactive oxygen species (ROS). Moreover, BMEVs downregulated the expression of NLRP3, which reduced the chemoresistance of OSCC cells to 5-FU.

## Results

### Preparation and cellular internalization of BMEVs

**Fig. 1:**
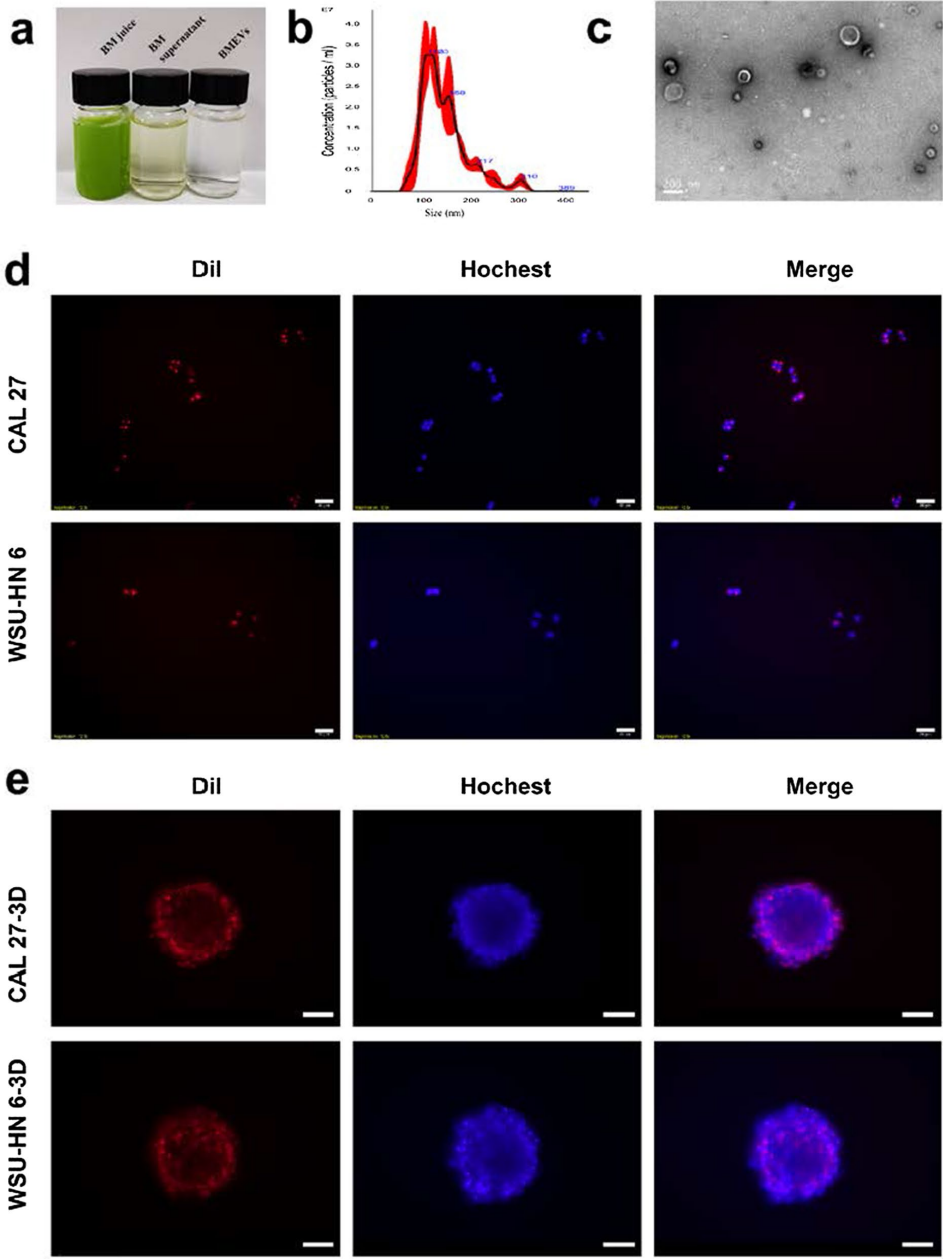
Preparation and cellular internalization of BMEVs. **a** Color of the BM juice, BM supernatant and BMEVs; **b** nanoparticle tracking analyzes the size and concentration of BMEVs; **c** transmission electron microscope images of BMEVs; **d** Fluorescence images of DiI-labeled BMEVs taken up by 2D cultured CAL 27 and WSU-HN6 cells, scale bar = 20 μm; **e** Fluorescence images of DiI-labeled BMEVs taken up by 3D cultured CAL 27 and WSU-HN6 cells, scale bar = 100 μm

BMEVs were isolated from bitter melon juice by electrophoresis- and dialysis-based methods. During separation, the volume showed no different changes (Fig. 1a), while the protein (Additional file 1: Figure S1A) and RNA (Additional file 1: Figure S1B) concentrations decreased significantly. Nanoparticle tracking analysis (NTA) showed the concentration and diameters of the BMEVs (Fig. 1b). To further characterize the isolated BMEVs, transmission electron microscopy (TEM) was performed. The intact and typical “cup-shaped” morphology of BMEVs was displayed, and the diameter was consistent with the NTA result (Fig. 1c). The proteomic analyses of BMEVs were also performed and the three protein (heat shock protein 70, *S*-adenosyl-homocysteinase and glyceraldehyde 3 phosphate dehydrogenase) markers for plant EVs were identified (Additional file 2: Table S3). Cellular internalization is the basic requirement of BMEVs to exert anticancer efficacy. The BMEVs were labeled with the lipophilic dye DiI (1,1'-dioctadecyl-3,3,3',3'-tetramethylindocarbocyanine perchlorate) to assess whether the BMEVs were internalized by OSCC cells. As shown in Fig. 1d, DiI-labeled BMEVs were observed in the CAL 27 and WSU-HN6 cells lines, and the cellular nuclei were stained with Hoechst. Three-dimensional (3D) spheroid culture was also investigated, as it can mimic solid tumors to some extent. The results indicated that BMEVs could also be efficiently taken up by 3D spheroid-cultured OSCC cells (Fig. 1e). These data demonstrated that BMEVs could be isolated from bitter melon juice and taken up by OSCC cells.

### Anti-proliferation effect of BMEVs

**Fig. 2:**
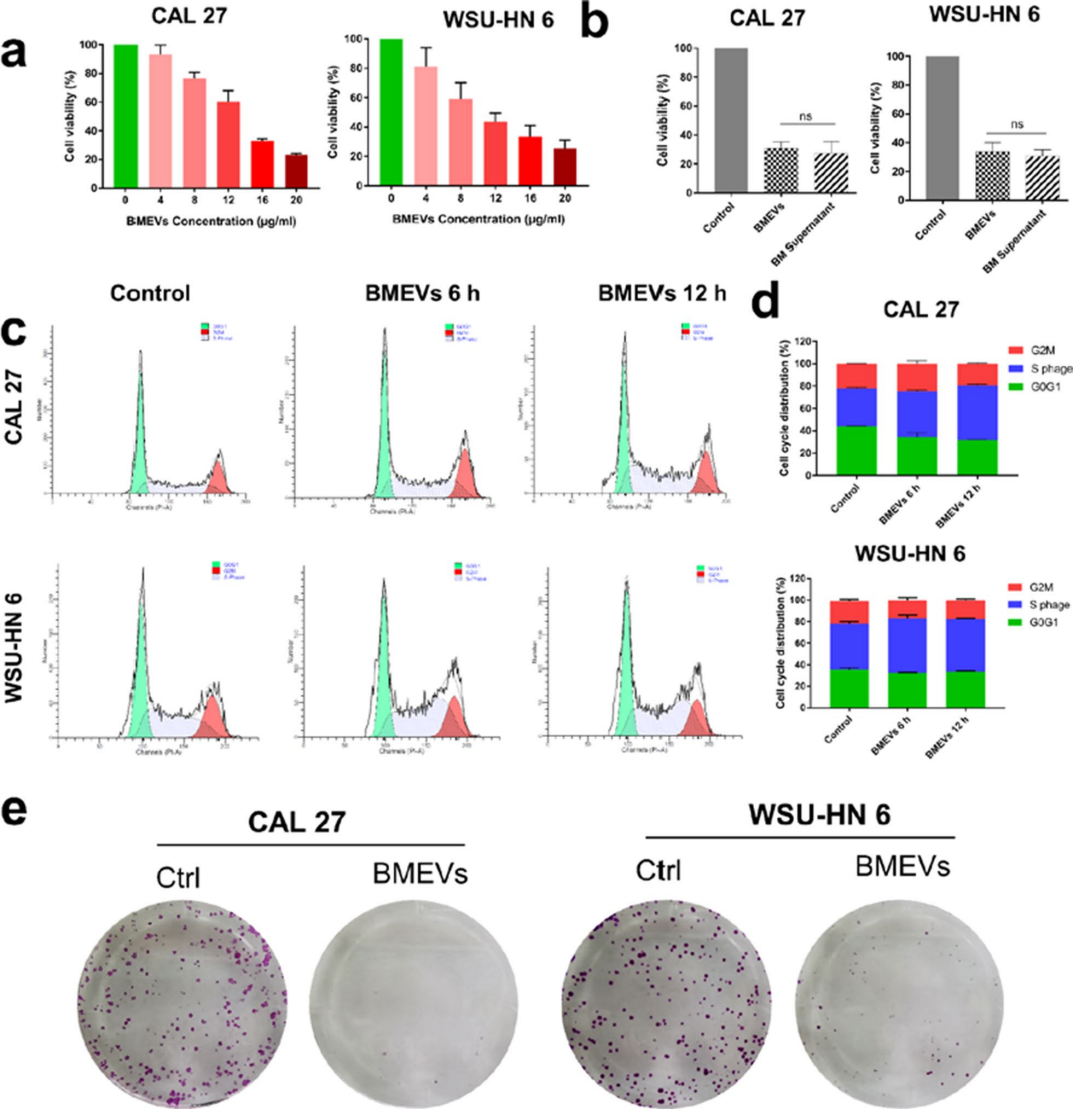
Anti-proliferation effect of BMEVs. **a** CCK-8 assay to evaluate the cell viability of CAL 27 and WSU-HN6 cells treated with different concentrations of BMEVs for 24 h; **b** cell viability of CAL 27 and WSU-HN6 cells treated with the same volume of BMEVs and BM supernatant; **c** flow cytometric analysis of cell-cycle phases after BMEVs treatment for 12 h; **d** representative column graph analysis of CAL 27 and WSU-HN6 cell cycle phases; **e** Plate colony formation assay of CAL 27 and WSU-HN6 cells

Several experimental approaches were used to detect the effect of BMEVs on OSCC. CCK-8 analysis revealed that BMEVs inhibited the proliferation of CAL 27 and WSU-HN6 cells in a dose-dependent manner (Fig. 2a). As the BMEVs were isolated from bitter melon juice, we wanted to investigate the anti-proliferation efficacy of the same volume of BMEVs and bitter melon supernatant. As shown in Fig. 2b, BMEVs achieved a similar inhibitory effect compared with bitter melon juice, which indicated that BMEVs played an essential role in the antitumor activity of bitter melon. To verify the details of cell inhibition, flow cytometer assays were performed (Fig. 2c), which demonstrated that BMEVs significantly induced S phase arrest (Fig. 2d and Additional file 1: Figure S2). Colony formation assays further displayed that BMEVs markedly suppressed the growth of OSCC cells (Fig. 2e). These results indicated that BMEVs could suppress the growth of OSCC.

### BMEVs induced the apoptosis of OSCC

**Fig. 3:**
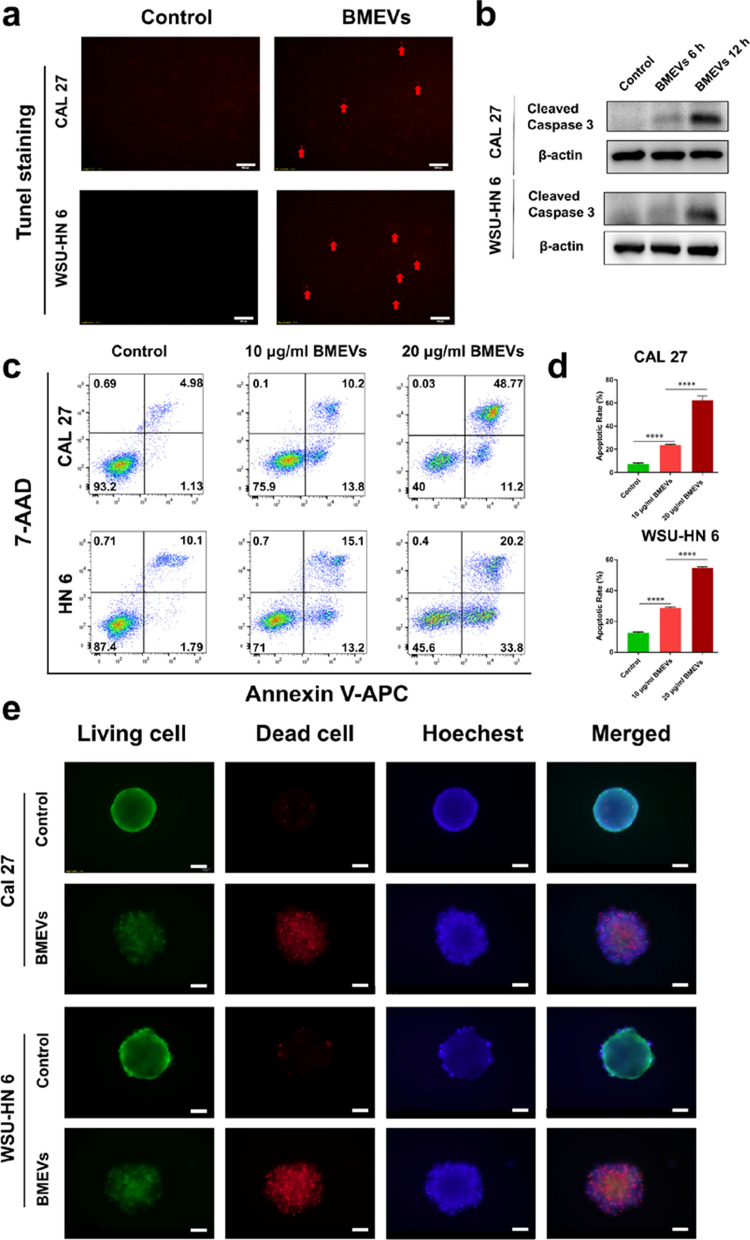
BMEVs induce the apoptosis of OSCC. **a** TUNEL staining assay of CAL 27 and WSU-HN6 cells after BMEVs treatment, scale bar = 100 μm; **b** Western blot analysis of cleaved caspase 3 protein expression; **c** CAL 27 and WSU-HN 6 cells were stained with Annexin-v and 7-AAD to analyze apoptosis rates by flow cytometry; **d** representative column graph analysis of the apoptosis rate; **e** fluorescence images of live/dead stained 3D cultured cells, scale bar = 100 μm

Then, we observed that there was a significant cell death induced by BMEVs. TUNEL staining detected the DNA breaks formed, which suggested that BMEVs could cause OSCC apoptosis (Fig. 3a). Western blot results confirmed that BMEVs exerted apoptotic effects on OSCC cells with upregulation of cleaved caspase 3 (Fig. 3b). Cell apoptosis was then determined by flow cytometry using Annexin-V and 7-AAD (7-Aminoactinomycin D) staining (Fig. 3c). Representative flow cytometry results are shown in Fig. 3d, which demonstrated that BMEVs caused massive cell apoptosis. The cytotoxicity of BMEVs was also displayed via live/dead cell co-staining in 3D-cultured OSCC cells. As shown in Fig. 3e, red fluorescence (dead cells) increased, while, green fluorescence (live cells) decreased after BMEVs treatment. These findings suggested that BMEVs could induce OSCC cells apoptosis.

### BMEVs stimulate ROS generation

**Fig. 4:**
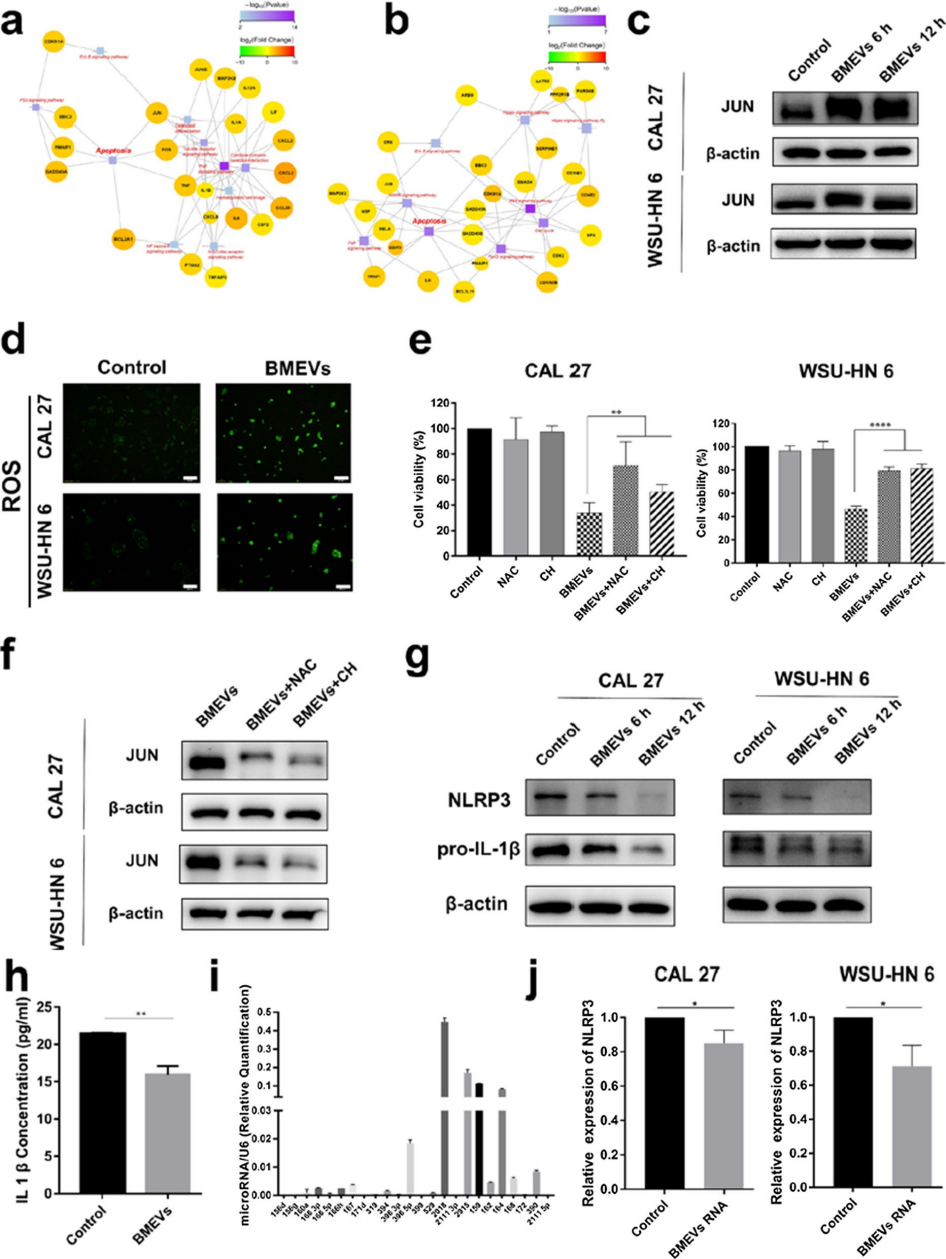
BMEVs stimulate ROS generation and downregulate NLRP3 expression. **a** KEGG network analysis of CAL 27 cells after BMEVs treatment at 6 h; **b** KEGG network analysis of CAL 27 cells after BMEVs treatment at 12 h; **c** Western blot analysis of JUN expression after BMEVs treatment; **d** fluorescence images of the intracellular ROS levels using DCFH-DA, scale bar = 200 μm; **e** cell viability of CAL 27 and WSU-HN 6 cells treated with BMEVs and scavenger NAC and CH; **f** Western blot analysis of JUN expression after BMEVs treatment with NAC or CH; **g** Western blot analysis of NLRP3 and pro-IL-1β expression after BMEVs treatment; **h** The IL-1β concentration of CAL27 cultured medium pre- and post-BMEVs treatment; **i** microRNAs extracted from BMEVs; **j** RT-PCR assay of relative expression of NLRP3 in CAL 27 and WSU-HN6 cells treated with BMEVs derived RNAs

To clarify the mechanism behind the apoptotic effect of BMEVs on OSCC cells, RNA sequencing was performed. Kyoto Encyclopedia Genes and Genomes (KEGG) network analysis was adopted to reveal the potential molecular mechanism and pathway. As shown in Fig. 4a, upregulated JUN expression was directly associated with apoptosis after BMEVs treatment for 6 h. JUN is also an important molecule that mediates the activation of the MAPK pathway, which was the top KEGG pathway after 12 h (Fig. 4b). Consistent with the gene expression pattern, upregulation of JUN protein was confirmed by Western blot (Fig. 4c). It is plausible that BMEVs may induce ROS production in OSCC cells, as the JUN protein is related to ROS stress. Then, the intracellular ROS level was detected. As shown in Fig. 4d a significant generation of ROS was observed at 12 h after BMEVs treatment. ROS have displayed double-edged sword properties in cancer treatment, as both pro- and antioxidant therapies have been proposed for cancer therapy. Thus, the function of generated ROS in OSCC cells should be determined. To this end, OSCC cells were pretreated with N-acetylcysteine (NAC) and catechin hydrate (CH), which are two inhibitors of ROS. Both the anti-proliferative effect and expression of JUN protein were significantly abrogated by NAC and CH (Fig. 4e and Fig. 4f). The results showed that BMEVs could stimulate the generation of ROS in OSCC cells and then induce the apoptosis of OSCC cells.

### BMEVs downregulate NLRP3 expression

Next, we evaluated the expression of NLRP3 as ROS could activate NLRP3, which is related to resistance to 5-FU. Surprisingly, BMEVs downregulated the gene expression of NLRP3 (Additional file 1: Figure S3) and protein expression of NLRP3 and pro-IL-1β (Fig. 4g). Consistent with the Western blot results, after BMEVs treatment, the IL-1β concentration in the CAL27 culture medium was decreased (Fig. 4h). Due to its low abundance, the IL-1β concentration of WSU-HN6 could not be detected. To determine the potential mechanism, total RNA was extracted from BMEVs. Twenty-four kinds of microRNAs were identified in BMEVs (Fig. 4i). The bioinformatic predictions indicate that 11 microRNAs have potential to regulate the expression of NLRP3 mRNA (Additional file 1: Tables S1 and S2). Quantitative RT-PCR analysis confirmed that the RNAs in BMEVs could downregulate the expression of NLRP3 (Fig. 4j). Consistent with the gene expression results, BMEVs derived RNAs downregulated the NLRP3 and pro-IL-1β protein expression (Additional file 1: Figure S4), which illustrated that BMEVs could downregulate the expression of NLRP3, at least in part, due to the RNAs in BMEVs.

### Synergistic effect of BMEVs and 5-FU on OSCC

**Fig. 5:**
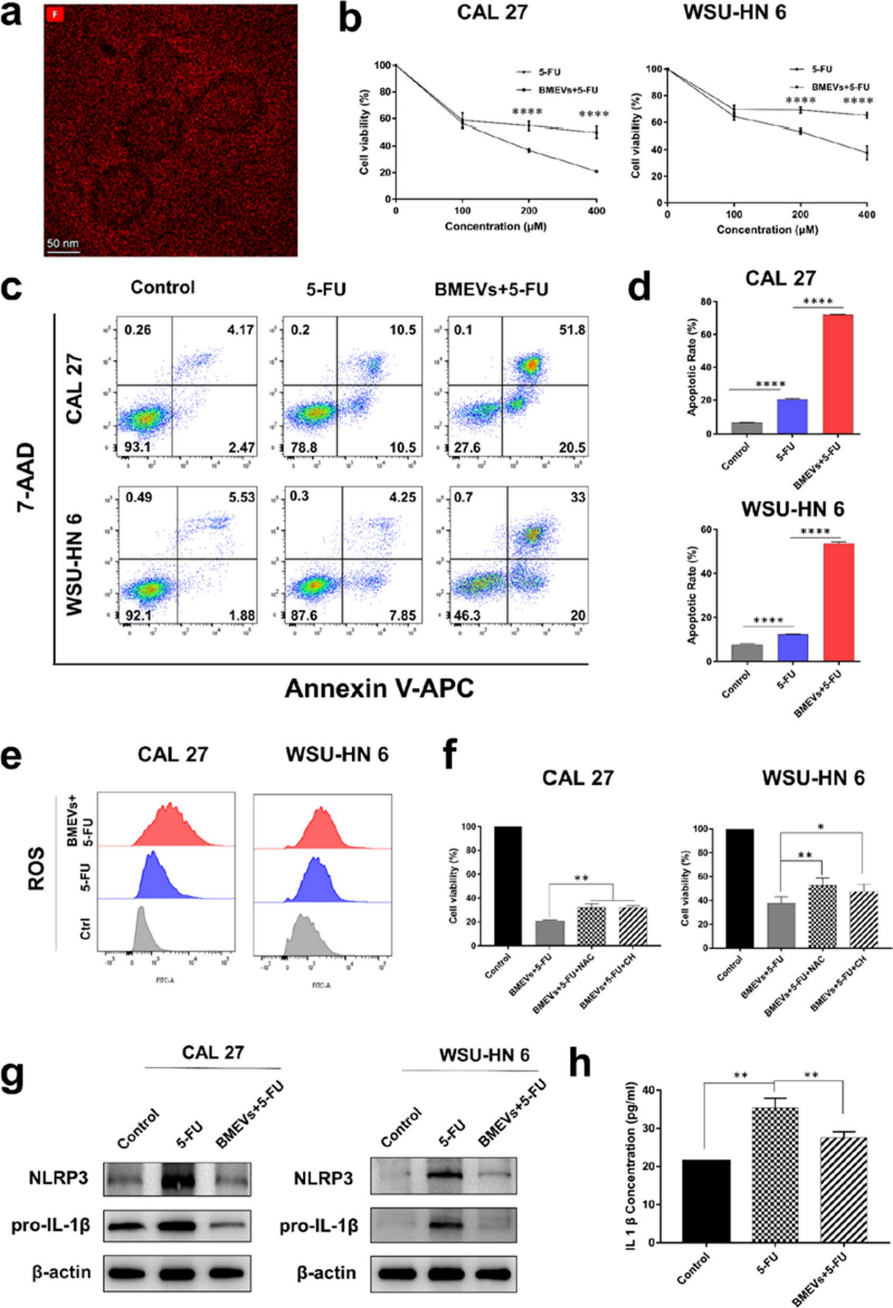
Synergistic effect of BMEVs and 5-FU on OSCC. **a** TEM image of fluorine element mapping; **b** CCK-8 assay to evaluate the cell viability of CAL27 and WSU-HN6 cells treated with different concentrations of 5-FU and BMEVs combined with 5-FU; **c** Flow cytometry analysis of the apoptosis of CAL 27 and WSU-HN 6 induced by 5-FU and BMEVs + 5-FU; **d** representative column graph analysis of the apoptosis rate; **e** Flow cytometry assay of the intracellular ROS levels; **f** Cell viability of CAL 27 and WSU-HN 6 cells treated with BMEVs + 5-FU and NAC or CH; **g** Western blot analysis of the NLRP3 and pro-IL-1β expression after 5-FU and BMEVs + 5-FU treatment; **h** The IL-1β concentration in CAL27 cultured medium

Considering the antitumor and anti-inflammatory bioactivities of BMEVs, we explored the therapeutic efficacy of BMEVs in combination with 5-FU. A TEM image of BMEVs + 5-FU is shown in Additional file 1: Figure S5, and element mapping of fluorine is displayed (Fig. 5a). The CCK8 assay demonstrated BMEVs enhanced the cytotoxicity of 5-FU (Fig. 5b). Flow cytometry assays confirmed that BMEVs + 5-FU increased the apoptotic rate of OSCC cells (Fig. 5c, d). Compared with free 5-FU, BMEVs + 5-FU induced more ROS production (Fig. 5e), which may be involved in the synergistic cytotoxicity effect. This hypothesis was verified as NAC and CH could significantly reverse the anti-proliferative effect (Fig. 5f). We next examined the expression of NLRP3, which is implicated in drug resistance. As shown in Fig. 5g, free 5-FU upregulated NLRP3 and pro-IL-1β expression. However, BMEVs + 5-FU downregulated the expression of the two proteins. Meanwhile, the concentration of IL-1β was also decreased after BMEVs + 5-FU treatment (Fig. 5h).

### BMEVs enhanced the cytotoxic effect of 5-FU in vivo

**Fig. 6:**
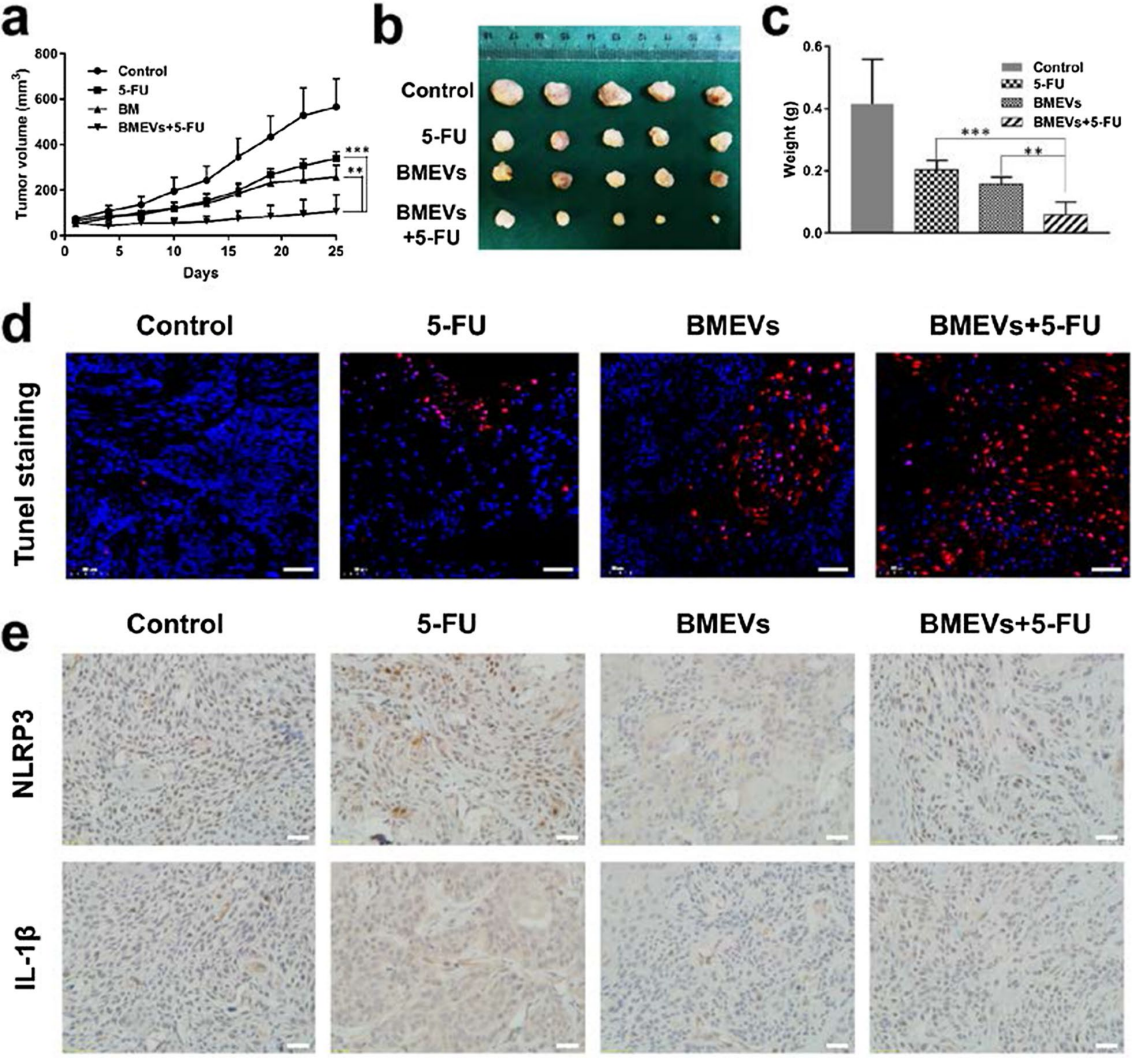
BMEVs enhanced the cytotoxic effect of 5-FU in vivo. **a** Tumor growth curve of the control, 5-FU, BMEVs and BMEVs + 5-FU groups; **b** Representative images of CAL 27 tumors; **c** Quantification analysis of the weight of the tumors; **d** Representative TUNEL staining images of the tumors, scale bar = 50 μm; **e** Immunohistochemistry images of NLRP3 and IL-1β, scale bar = 200 μm

To further evaluate the synergistic efficacy of BMEVs, OSCC tumor models were established. Mice were divided into four groups. As shown in Fig. 6a, the tumor volumes were calculated, and the growth of the tumor was the slowest in the BMEVs + 5-FU group. At the end of treatment, the mice were sacrificed and the tumors were collected and imaged (Fig. 6b). The tumor weights were recorded, and the tumors from BMEVs + 5-FU group were significantly lighter than those from the 5-FU and BMEVs groups (Fig. 6c). TUNEL staining of tumor sections demonstrated that more apoptotic tumor cells were observed in the BMEVs + 5-FU group (Fig. 6d). NLRP3 and IL-1β expression were also evaluated. As shown in Fig. 6e, decreased expression of NLRP3 and IL-1β was found in tumors derived from the BMEVs + 5-FU group compared with those from the 5-FU group. These data suggested that BMEVs could improve the therapeutic efficacy while reducing the drug resistance of 5-FU.

## Discussion

Recently, edible plant-derived extracellular vesicles (PDEVs) have emerged as therapeutics [2, 16, 17]. Bitter melon is cultivated as a vegetable and folk medicine, and has been reported to possess anticancer and anti-inflammatory activities [18, 19]. An extract of bitter melon has inhibited cell proliferation in several cancer lines including breast cancer cells [20], prostate cancer [21], pancreatic cancer [22], and OSCC [14, 23]. However, the role of bitter melon derived extracellular vesicles (BMEVs) in OSCC has never been described. In the present work, we first isolated and characterized BMEVs from bitter melon juice. BMEVs could be taken up by OSCC cells and they induced the apoptosis of the cells. More importantly, BMEVs achieved similar therapeutic effect compared with the same volume of bitter melon juice, even though the protein and RNA concentration of the BM juice was over 100-fold that of BMEVs, which implies the powerful antitumor bioactivity of BMEVs. Chemoprevention with natural products has potential applications in cancer treatment [24–27]. A recent study reported that bitter melon extracts induced the apoptosis of OSCC cell lines via ROS-mediated mitochondrial injury [28]. The mechanism behind the growth suppression of OSCC cells was also related to ROS generation as NAC and CH abrogated the antitumor efficacy of BMEVs. Our results suggest that extracellular vesicles could serve as a natural product for chemoprevention.

Recently, Marcela Pinedo et al. have identified three protein families commonly found amongst plants EVs. They are heat shock protein 70 (HSP70), S-adenosyl-homocysteinase and glyceraldehyde 3 phosphate dehydrogenase [29]. In this study, we performed the proteomics analyses of BMEVs and found all the three proteins, which further confirmed the purification of BMEVs. Meanwhile, MAP30 has been found in BMEVs. MAP30 could increase the intracellular Ca^2+^ ion concentration, which triggered ROS-mediated cancer cell death via apoptosis [30]. In our study, we observed the generation of ROS after BMEVs treatment, which may mediate by MAP30 protein in BMEVs.

Inflammation is a critical component of tumor progression [31]. Chemotherapy-enhanced inflammation may lead to the failure of therapy and metastasis [10]. The NLRP3 inflammasome is one of the critical components of the innate immune system and it plays an important role in cancers [32, 33]. Many factors can activate NLRP3 inflammasomes including K^+^ efflux, intracellular calcium, endoplasmic reticulum (ER) stress, and ROS [34]. Our previous findings revealed that 5-FU treatment increased the expression of NLRP3 in OSCC, which mediated drug resistance [11]. We have also proven that NLRP3 could promote tumor growth and metastasis in OSCC [35]. It has been reported that microRNA22 could repress cancer cell growth by downregulating NLRP3 in our and other previous studies [36, 37]. Thus, it is necessary to clarify the expression of NLRP3 status after BMEVs treatment as ROS could stimulate NLRP3 expression, which promotes the growth of OSCC [35, 38]. Surprisingly, BMEVs inhibited NLRP3 expression even during ROS generation. In previous studies, ginger- and mushroom-derived EVs downregulated the expression of NLRP3 [4, 5]. Previous research indicated that plant miRNAs could regulate the expression of target genes in animals and contribute to the function of tissues [39]. Conserved bitter melon microRNAs have been identified [40]. In this study, twenty-four kinds of microRNAs were detected in BMEVs. The bioinformatic predictions indicate that 11 microRNAs have potential to regulate the expression of NLRP3 mRNA, and BMEVs derived RNAs could downregulate the protein expression of NLRP3. In future work, more attention should be given to exploring which RNAs in BMEVs mediate their anti-inflammatory function.

5-FU could induce mitochondrial ROS leading to cancer cell death [41]. Elevated ROS production enhanced the antitumor effects of 5-FU on hepatocellular carcinoma [42]. Antioxidants decreased the apoptotic effect of 5-FU in colon cancer [43]. In this study, BMEVs combined with 5-FU enhanced OSCC apoptosis via increased ROS generation. Although 5-FU is beneficial in OSCC therapy, the resistance of 5-FU by tumor cells limits its clinical application. Our previous study suggested that 5-FU treatment upregulated NLRP3 expression which mediated the chemoresistance of OSCC to 5-FU [11]. Many natural products have been proven to prevent cancer and play roles in reducing chemoresistance [44]. In this research, we demonstrated that BMEVs could enhance the cytotoxic effects and reduce the drug resistance of 5-FU during OSCC treatment by downregulating the expression of NLRP3. Extracellular vesicles possess several characteristics that qualify them as promising vehicles for drug delivery [45]. Surface modification will facilitate the application of EVs. Cell penetrating peptides (CPPs) were widely used for drug delivery [46–48]. Folic acid-modified ginger derived EVs have been used for advanced drug-delivery [49]. Considering the location of oral cancer, in this study BMEVs + 5-FU were peritumorally administrated. For further exploration, the tumor-targeting ability of BMEVs should be clarified. With continued development, PDEVs will become a viable alternative drug-delivery vehicle with intrinsic bioactivities for cancer treatment.

## Materials and methods

### Cell culture

The human OSCC cell line CAL 27 was obtained from the American Type Culture Collection and WSU-HN6 was obtained from the National Institutes of Health. The cells were cultured in Dulbecco’s modified Eagle’s medium supplemented with 10% fetal bovine serum and 1% penicillin–streptomycin solution. Three-dimensional (3D) spheroid cultured CAL 27 and WSU-HN6 was also performed. Briefly, 2000 cells/well in 100 μl of medium containing 10% FBS and supplemented with 0.25% methylcellulose solution were seeded in 96-well U-shaped bottom plates and were cultured under standard conditions. The cells were cultured in a humidified atmosphere of 5% CO_2_ at 37 °C.

### Bitter melon juice preparation

Bitter melons were purchased from a local market and squeezed to obtain the juice. The juice was sequentially centrifuged at 3000×*g* for 10 min, and 10,000×*g* for 20 min. The supernatant was filtered through a 0.22 μm pore filter for isolation of BMEVs.

### BMEVs isolation

BMEVs were isolated by electrophoresis- and dialysis-based methods as described in our previous report [3]. In detail, five milliliters of filtered bitter melon supernatant was loaded into a 300 kDa dialysis bag. The dialysis bag was placed in a gel holder cassette under a current of 300 mA. After 30 min, the electrophoretic buffer was replaced, and the electrophoretic direction was changed. After 2 h, the purified BMEVs were collected.

### Combination of BMEVs with 5-FU

The BMEVs and 5-FU mixture was sonicated for 10 min in sonic cleaner followed by a five-minute cooling period.

### Transmission electron microscopy (TEM)

Five milliliters of BMEVs were concentrated to 200 μl through 100 kDa ultrafiltration. Concentrated BMEVs (5 μl) were added to 200 mesh carbon-coated grids for 1 min. Then, the grids were dried with filter paper. For negative staining, 5 μl of 2% uranyl acetate was dropped onto the grids. After 1 min, the excess negative staining solution was removed. The samples were viewed with a transmission electron microscope. BMEVs combined with 5-FU and the corresponding element mapping of fluorine were also detected with TEM.

### Nanoparticle tracking analysis (NTA)

Quantification and size determination of BMEVs were assessed by using a NanoSight NS500 instrument (Malvern). The instrument was set up to operate at room temperature. Three videos were recorded for each specimen, and the results were analyzed with NTA software.

### Proteomics analyses

BMEVs were concentrated with 100 kDa ultrafiltration then Sangon Technology subjected it to proteomics analyses.

### Cellular uptake

CAL 27 and WSU-HN6 cells were seeded in a 6-well plate for 2D culture and a 96-well plate for 3D culture. DiI-labeled BMEVs were then added to the wells. After incubation for 6 h at 37 °C, the cells were stained with Hoechst for 5 min. Then, the cells were washed three times with PBS and observed with fluorescence microscopy or analyzed by flow cytometry.

### Viability assay

Cell viability was assessed with Cell Counting Kit-8 (CCK-8) assay. Briefly, CAL 27 (1.5 × 10^4^) and WSU-HN6 (0.5 × 10^4^) cells were seeded in 96-well plates and exposed to different doses of BMEVs, 5-FU, and BMEVs combined with 5-FU for 24 h. N-acetyl cysteine (5 mM) or catechin hydrate (100 μg/ml) was also added to inhibit the generation of ROS.

### Plate colony formation assay

A total of 800 CAL 27 and WSU-HN6 cells were cultured in 6-well plates. After 24 h, the culture medium was replaced with fresh cell culture medium supplemented with BMEVs, and the cells were cultured for 2 weeks. The colonies were fixed and stained with 0.1% crystal violet.

### Flow cytometry

Flow cytometry was used to evaluate the cell cycle. CAL 27 and WSU-HN6 cells were treated with 20 μg/ml BMEVs for 6 and 12 h. The cells were collected and fixed with 70% ethanol at − 20 °C overnight before propidium iodide (PI) staining for flow cytometry analysis.

Flow cytometry was used to discriminate between intact and apoptotic cells. CAL 27 and WSU-HN6 cells were treated with BMEVs for 24 h, and analyzed by flow cytometry.

### Western blot

Primary antibodies against cleaved caspase 3, JUN, NLRP3, pro-IL-1β, and β-actin were used to detect the target proteins at 4 °C overnight. The proteins were incubated with HRP-labeled secondary antibodies at room temperature for 1 h and visualized with an enhanced chemiluminescence detection system.

### TUNEL staining

CAL 27 and WSU-HN6 cells were cultured in 6-well plates and pretreated with 20 μg/ml BMEVs for 12 h. TUNEL staining was performed according to the manufacturer’s instructions.

### Live-dead assay

3D cultured spheroids of CAL 27 and WSU-HN6 cells were pretreated with 20 μg/ml BMEVs for 24 h. Then, calcein-AM and PI were co-cultured with the cells for 10 min. The spheroids were washed three times with PBS and observed with fluorescence microscopy.

### RNA sequencing

CAL 27 cells were treated with BMEVs for 6 or 12 h. Total RNA was extracted using TRIzol reagent, and then Sangon Technology subjected it to RNA sequencing and data analysis.

### Quantitative real-time PCR (qRT-PCR)

RNAs were extracted from concentrated BMEVs using TRIzol reagent and Dr. GenTLE™ Precipitation Carrier (Takara; Code No. 9094). The microRNAs were detected with miRcute Plus miRNA qPCR Detection Kit (TIANGEN; PF411). The primers used were displayed in Additional file 1: Table S1.

CAL 27 and WSU-HN6 cells were cultured in 6-well plates and treated with 300 ng/ml BMEVs RNAs. After 12 h, total RNA was extracted from CAL 27 and WSU-HN6. The relative expression of NLRP3 was detected by qRT-PCR following the manufacturer’s instructions. The primers used were as follows: NLRP3: forward 5’-CCATCGGCAAGACCAAGA-3’, reverse 5’-ACAGGCTCAGAATGCTCATC-3’; GAPDH: forward 5’-GTATCGTGGAAGGACTCATGAC-3’, reverse 5’- ACCACCTTCTTGATGTCATCAT-3’.

### Reactive oxygen species (ROS) detection

The cellular ROS level was detected by 2',7'-dichlorofluorescein diacetate (DCFH-DA). CAL 27 and WSU-HN6 cells were pretreated with BMEVs, 5-FU, and BMEVs + 5-FU for 12 h. DCFH-DA (10 μmol) was cultured with the cells for 20 min at 37 °C. DCFH-DA was observed with fluorescence microscopy or analyzed by flow cytometry.

### IL-1β measurement

CAL 27 cells were cultured in 6 cm plates and pretreated with BMEVs, 5-FU and BMEVs combined with 5-FU for 12 h. The cultured media were collected and measured with an electrochemiluminescence assay.

### Animals and tumor model

Animal studies were performed in 4- to 6-week-old female BALB/c nude mice purchased from the Shanghai Laboratory Animal Center. All mice were housed under pathogen-free conditions in the animal care facilities of Shanghai Ninth People’s Hospital, Shanghai Jiao Tong University School of Medicine. A total of 2 × 10^6^ CAL 27 cells were injected into the flanks of the nude mice. When the average tumor volume reached approximately 50 mm^3^, the mice were injected peritumorally with 50 mg/kg 5-FU, BMEVs + 5-FU, the same volume of BMEVs and control electrophoretic buffer solution every 3 days four times in total. After 24 days, the mice were sacrificed, and the tumors were weighed. The tumor volumes were measured twice per week and calculated using the following formula: length × width^2^/2.

### Immunohistochemistry

Formalin-fixed, paraffin-embedded tumors were subjected to staining and immunohistochemistry using primary antibodies against NLRP3 or IL-1β and observed with microscopy.

### Statistical analysis

The results expressed as the mean ± standard deviation were carried out in triplicate. Graphpad Prism 7 software was used to conduct the statistical analyses of the data. This analysis was performed using Student’s t-test since the data followed a normal distribution. *P < 0.05; **P < 0.005; ***P < 0.001; ****P < 0.0001; ns, not significant.

## Supplementary Information

**Additional file 1: Figure S1.** (A) The protein and (B) RNA concentration of BM supernatant and BMEVs. **Figure S2.** S phage of CAL 27 and WSU-HN6 after BMEVs treatment. **Figure S3.** RT-PCR assay of the relative expression of NLRP3 in CAL 27 and WSU-HN6 cells treated with BMEVs. **Figure S4.** Western blot analysis of NLRP3 and pro-IL-1β expression after BMEVs associated RNAs treatment. **Figure S5.** TEM image of BMEVs combined with 5-FU. **Table S1.** Primer used to detect BMEVs derived microRNAs. **Table S2.** microRNAs have potential to regulate NLRP3 mRNA.

**Additional file 2: Table S3.** The proteins of BMEVs.

## Data Availability

The datasets used and/or analyzed during the current study are available from the corresponding author on reasonable request.

## Abbreviations

EVs: Extracellular vesicles
PDEVs: Plant derived extracellular vesicles
BMEVs: Bitter melon extracellular vesicles
5-FU: 5-Fluorouracil
OSCC: Oral squamous cell carcinoma
NLRP3: NOD-like receptor family pyrin domain containing 3
NTA: Nanoparticle tracking analysis
TEM: Transmission electron microscope
3D: Three-dimensional
DiI: Dioctadecyl-3,3,3,3-tetramethylindodicarbocyanine
7-AAD: 7-Aminoactinomycin D
NAC: *N*-Acetylcysteine
CH: Catechin hydrate CH
ROS: Reactive oxygen species
DCFH-DA: 2',7'-Dichlorofluorescin diacetate
H&E: Hematoxylin and eosin
