# Supplementary material for: Bitter melon derived extracellular vesicles enhance the therapeutic effects and reduce the drug resistance of 5-fluorouracil on oral squamous cell carcinoma
Source: J Nanobiotechnology. 2021 Aug 28;19:259. doi: 10.1186/s12951-021-00995-1 (PMC8400897; doi:10.1186/s12951-021-00995-1)
Supplement: Supplementary file 1 — Additional file 1: Figure S1. (A) The protein and (B) RNA concentration of BM supernatant and BMEVs. Figure S2. S phage of CAL 27 and WSU-HN6 after BMEVs treatment. Figure S3. RT-PCR assay of the relative expression of NLRP3 in CAL 27 and WSU-HN6 cells treated with BMEVs. Figure S4. Western blot analysis of NLRP3 and pro-IL-1β expression after BMEVs associated RNAs treatment. Figure S5. TEM image of BMEVs combined with 5-FU. Table S1. Primer used to detect BMEVs derived microRNAs. Table S2. microRNAs have potential to regulate NLRP3 mRNA. [file 12951_2021_995_MOESM1_ESM.docx]

**Bitter melon derived extracellular vesicles enhance the therapeutic effects and reduce drug resistance of 5-fluorouracil on** **oral squamous cell carcinoma**

Meng Yang ^1^, Qingqiong Luo^1^, Xu Chen ^1^, and Fuxiang Chen^1,2,^*

^1^ Department of Clinical Immunology, Ninth People’s Hospital, Shanghai Jiao Tong University School of Medicine, Shanghai, 200011, P. R. China

^2^ Faculty of Medical Laboratory Science, School of Medicine, Shanghai Jiao Tong University, Shanghai, 200025, P. R. China.

***** Correspondence: [chenfx@sjtu.edu.cn](mailto:chenfx@sjtu.edu.cn)


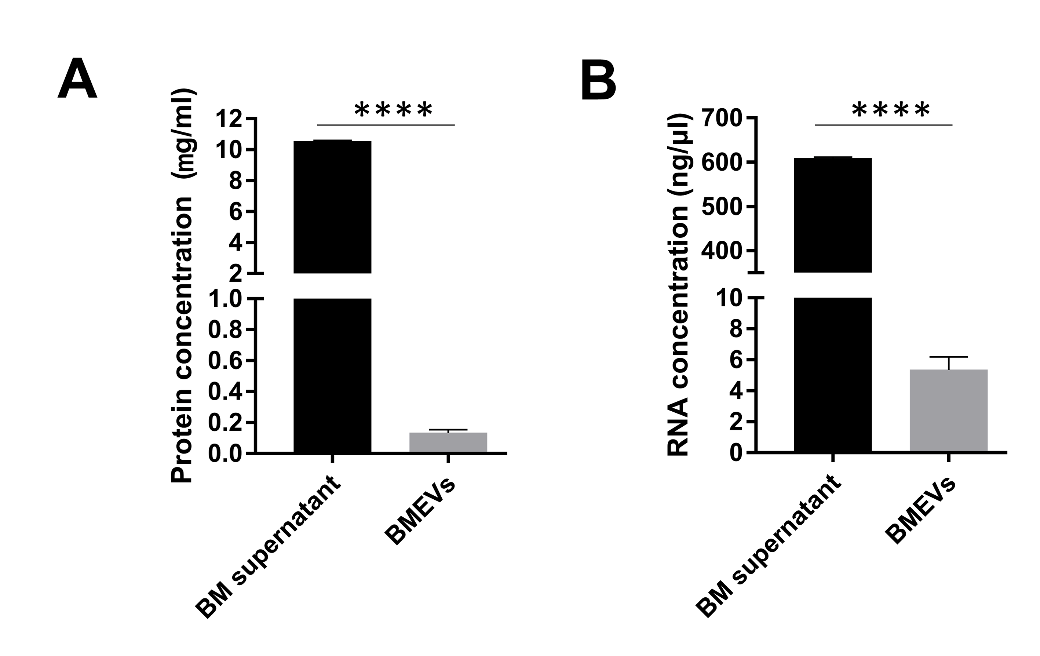


Figure S1. (A) The protein and (B) RNA concentration of BM supernatant and BMEVs


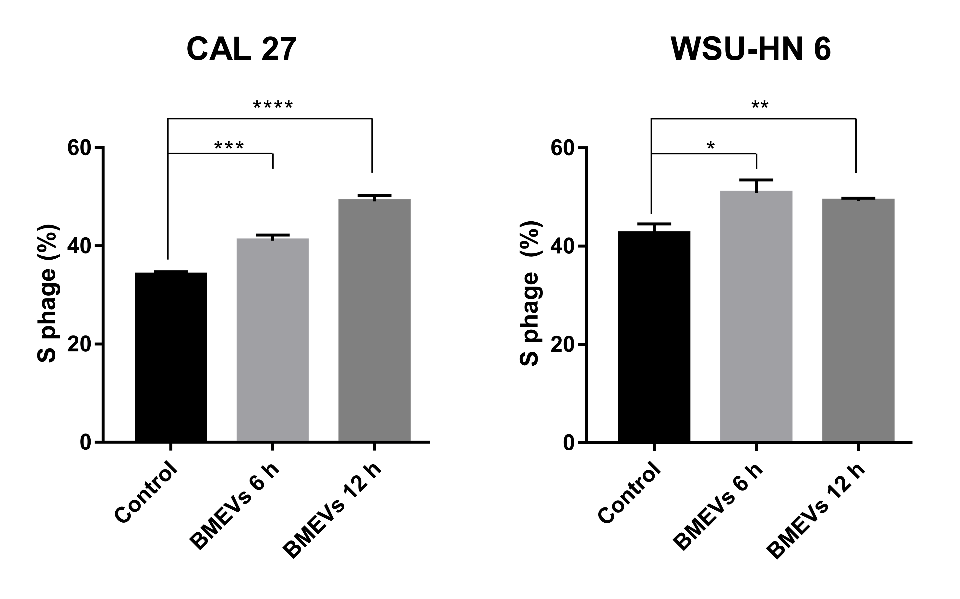


Figure S2. S phage of CAL 27 and WSU-HN6 after BMEVs treatment


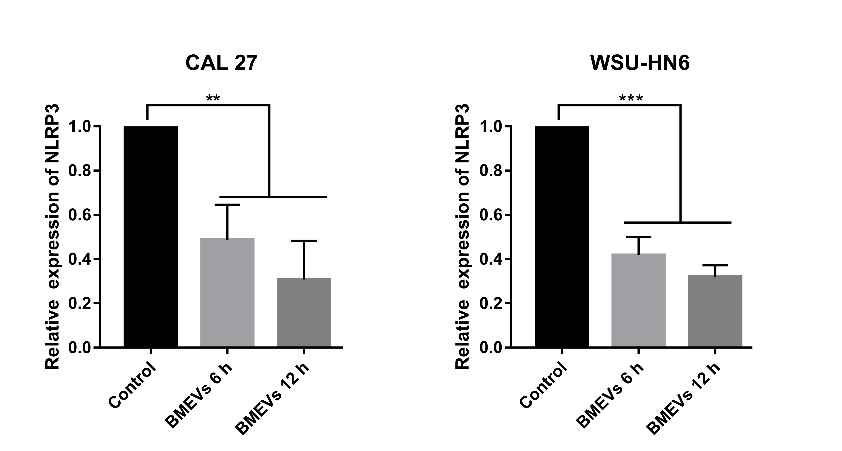


Figure S3. RT-PCR assay of the relative expression of NLRP3 in CAL 27 and WSU-HN6 cells treated with BMEVs


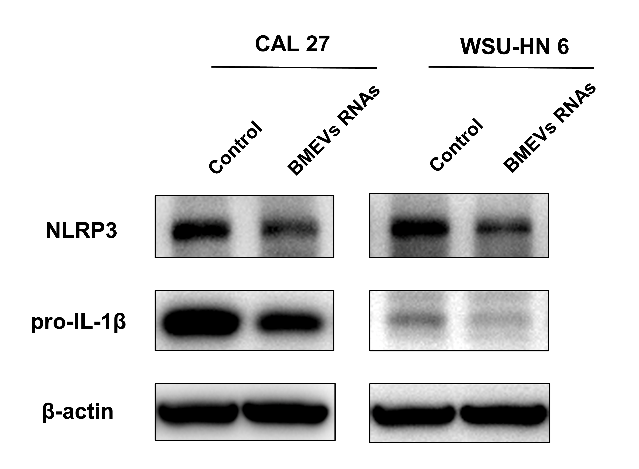


Figure S4. Western blot analysis of NLRP3 and pro-IL-1β expression after BMEVs associated RNAs treatment


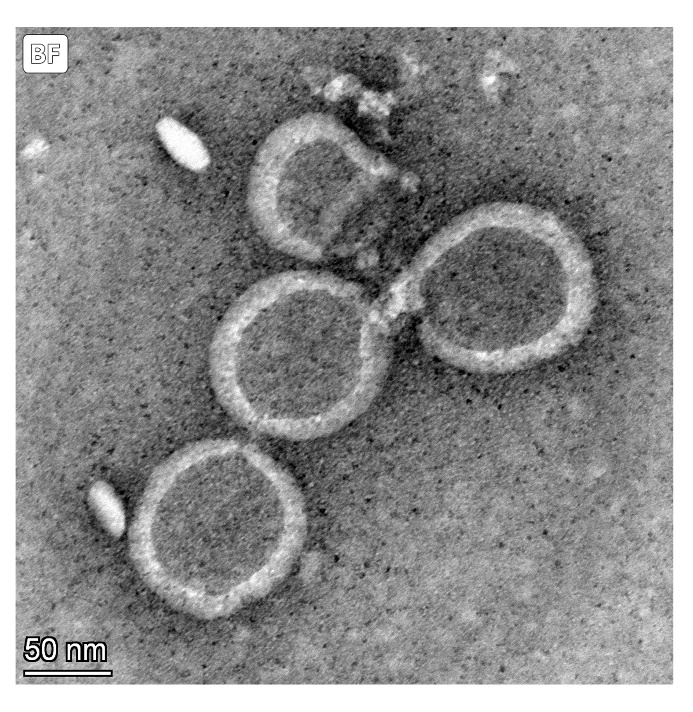


Figure S5. TEM image of BMEVs combination with 5-FU

Table S1. Primer used to detect BMEVs derived microRNAs

| **microRNA** | **Forward Primer** |
| --- | --- |
| 156d | TGACAGAAGAGAGTGAGCAC |
| 156g | CGGTGACAGAAAGAGAGAAAGCAC |
| 159 | TTTGGATTGAAGGGAGCTC |
| 160a | TGCCTGGCTCCCTGTAT |
| 162 | TCGATAAACCTCTGCATCCAG |
| 164d | TGGAGAAGCAGGGCA |
| 166 3p | TCGGACCAGGCTTCATT |
| 166 5p | GGAATGTTGTCTGGCTCGAGG |
| 166h | TCGGACCAGGCTTCATTCCCC |
| 167 | TGAAGCTGCCAGCATGATCTA |
| 168 | TCGCTTGGTGCAGGTCGAA |
| 171 | TTGAGCCGTGCCAATATCACG |
| 172 | AGAATCTTGATGATGCTGC |
| 319 | TTGGACTGAAGGGAGCTCCT |
| 390 | AAGCTCAGGAGGGATAGCG |
| 394 | TTGGCATTCTGTCCACCTCC |
| 396 5p | TTCCACAGCTTTCTTGAAC |
| 396 3p | GCTCAAGAAAGCTGTGGGACA |
| 399d | TGCCAAAGGAGAGTTGCCCT |
| 529 | AGAAGAGAGAGAGCACAACCC |
| 2018 | GCCCGTCTAGCTCAGTTGGT |
| 2111 5p | TAATCTGCATCCTGAGGCG |
| 2111 3p | GCCCTCGGGTTGCAGATTA |
| 2915 | CCCGTCTAGCTCAGTTGGTA |
| U6 | CTCGCTTCGGCAGCACA |

Table S2. microRNAs have potential to regulate NLRP3 mRNA

| **microRNA** | **CT Value** |
| --- | --- |
| 156 d | 29 |
| 162 | 25 |
| 166 5p | 28 |
| 167 | 27 |
| 172 | 28 |
| 390 | 24 |
| 394 | 26 |
| 396 3p | 28 |
| 399 | 29 |
| 529 | 27 |
| 2111 5p | 28 |
